# Plasma Levels of Transforming Growth Factor-β1 Reflect Left Ventricular Remodeling in Aortic Stenosis

**DOI:** 10.1371/journal.pone.0008476

**Published:** 2009-12-30

**Authors:** Ana V. Villar, Manuel Cobo, Miguel Llano, Cecilia Montalvo, Francisco González-Vílchez, Rafael Martín-Durán, María A. Hurlé, J. Francisco Nistal

**Affiliations:** 1 División de Farmacología, Facultad de Medicina, Universidad de Cantabria, Instituto de Formación e Investigación Marqués de Valdecilla, Santander, Cantabria, Spain; 2 Servicio de Cardiología, Hospital Universitario Marqués de Valdecilla, Instituto de Formación e Investigación Marqués de Valdecilla, Santander, Cantabria, Spain; 3 Servicio de Cirugía Cardiovascular, Hospital Universitario Marqués de Valdecilla, Instituto de Formación e Investigación Marqués de Valdecilla, Santander, Cantabria, Spain; Harvard Medical School, United States of America

## Abstract

**Background:**

TGF-β1 is involved in cardiac remodeling through an auto/paracrine mechanism. The contribution of TGF-β1 from plasmatic source to pressure overload myocardial remodeling has not been analyzed. We investigated, in patients with valvular aortic stenosis (AS), and in mice subjected to transverse aortic arch constriction (TAC), whether plasma TGF-β1 relates with myocardial remodeling, reflected by LV transcriptional adaptations of genes linked to myocardial hypertrophy and fibrosis, and by heart morphology and function.

**Methodology/Principal Findings:**

The subjects of the study were: 39 patients operated of AS; 27 healthy volunteers; 12 mice subjected to TAC; and 6 mice sham-operated. Myocardial samples were subjected to quantitative PCR. Plasma TGF-β1 was determined by ELISA. Under pressure overload, TGF-β1 plasma levels were significantly increased both in AS patients and TAC mice. In AS patients, plasma TGF-β1 correlated directly with aortic transvalvular gradients and LV mass surrogate variables, both preoperatively and 1 year after surgery. Plasma TGF-β1 correlated positively with the myocardial expression of genes encoding extracellular matrix (collagens I and III, fibronectin) and sarcomeric (myosin light chain-2, β-myosin heavy chain) remodelling targets of TGF-β1, in TAC mice and in AS patients.

**Conclusions/Significance:**

A circulating TGF-β1-mediated mechanism is involved, in both mice and humans, in the excessive deposition of ECM elements and hypertrophic growth of cardiomyocytes under pressure overload. The possible value of plasma TGF-β1 as a marker reflecting preoperative myocardial remodeling status in AS patients deserves further analysis in larger patient cohorts.

## Introduction

Aortic valve stenosis (AS) is one of the most common valvular diseases whose prevalence is likely to rise in the future with the increase in life expectancy of the population [1]. This entity promotes a pure, progressive, LV pressure overload, which is responsible for biomechanical stress, alterations in the humoral cell environment, and activation of numerous signaling pathways, bringing about structural and functional changes in the myocardium. Typically, LV remodeling under pressure overload is characterized by hypertrophic growth of cardiomyocytes, proliferation of cardiac fibroblasts, increased deposition of extracellular matrix (ECM) constituents and loss of myocytes with fibrotic replacement [2]. The major long-term consequences of this remodeling process are increased myocardial stiffness and decreased compliance resulting, initially, in diastolic dysfunction and, over time, in combined diastolic and systolic heart failure.

Abundant experimental evidence indicates that Transforming Growth Factor-β1 (TGF-β1) is critically involved in major structural changes of the myocardium under pressure overload [3], [4]. TGF-β1 acts on cardiomyocytes as well as on cardiac fibroblasts inducing hypertrophy of the former, synthesis of ECM materials by the latter [4] and endothelial-to-mesenchymal transition that recruits fibroblasts to the myocardium [5]. In addition, TGF-β1 is critically involved in phenotypic modulation of fibroblasts into matrix-producing myofibroblasts [6].

Few studies have dealt with such a role for TGF-β1 in human hypertrophied myocardium from pressure-overload related heart pathologies [7], including AS [8], [9]. Other groups and ours have previously reported increased myocardial expression (at the mRNA and/or protein levels) of TGF-β1 in patients with severe AS, together with upregulation of collagens I and III and fibronectin, as well as the sarcomeric protein myosin light chain-2 [8], [9]. Our results also support a regulatory role for myocardial TGF-β1 in the transcriptional activity of these remodeling-related genes, mediated by both the SMAD and the TGF-β activated kinase 1 (TAK1) signaling pathways [9].

The available experimental reports are focused in analyzing the autocrine and/or paracrine effects of TGF-β1 released by cardiomyocytes [10] and fibroblasts [11]. In the present study, we investigated, in mice subjected to aortic arch constriction and in patients with severe valvular AS, the contribution of TGF-β1 from circulating source to pressure overload-induced myocardial remodeling, as reflected by LV transcriptional changes of genes linked to myocardial hypertrophy and fibrosis, and by changes in echocardiographic heart morphology and function.

## Results

### Demographics and Clinical Characteristics of Patients

The characteristics of the cohorts are shown in Table 1. There were no significant differences between control and AS groups in mean age and incidence of atrial fibrillation-flutter. Concerning LV hypertrophy related factors, the mean body mass index and the incidence of obesity and diabetes did not differ between AS men and women and their control pairs. A history of systemic hypertension and the use of diuretics were more frequent among AS women than their controls, but the average systolic and diastolic blood pressures were higher in the control group. There were no other significant differences in the pharmacologic treatment with ACE inhibitors, angiotensin-II receptor antagonists, diuretics (in men), calcium antagonists, statins or β-blockers between the groups.

**Table 1:** Characteristics of patients.

	Control		Aortic Stenosis	
	Men(n = 14)	Women(n = 13)	Men(n = 21)	Women(n = 18)
**Age (yrs±SEM)**	71.9±1.6	69.1±2.2	70.3±3.0	72.6±2.6
**Systemic hypertension, n (%)**	3 (21)	1 (8)	11 (52)	9 (50) *
**Body Mass Index, kg/m^2^**	27.6±0.7	26.8±1.1	28.0±1.0	29.2±1.4
**Body Mass Index ≥30, n (%)**	2 (14)	3 (23)	5 (24)	7 (39)
**Diabetes Mellitus**	0	0	6 (29)	2 (11)
**ACE inhibitors, n (%)**	2 (14)	3 (25)	6 (29)	2 (11)
**AT-II receptor antagonists, n (%)**	1(7)	1 (8)	1 (5)	2 (11)
**Diuretics, n (%)**	2 (14)	2 (17)	5 (24)	10 (56) *
**Calcium antagonists, n (%)**	1 (7)	1 (8)	0	4 (22)
**β-Blockers, n (%)**	1 (7)	2 (17)	2 (10)	2 (11)
**Statins, n (%)**	5 (36)	1 (8)	2 (10)	5 (28)
**Atrial fibrillation or flutter, n (%)**	1 (7)	0	3 (14)	4 (22)
**Systolic blood pressure, mm Hg**	121.3±9.7	142.6±6.3	118.1±4.7	120.4±4.5 *
**Diastolic blood pressure, mm Hg**	69.2±5.2	82.6±2.0	64.9±2.4	66.1±3.4 *
**Peak AV gradient, mm Hg**	–	–	103±7	110±5
**Mean AV gradient, mm Hg**	–	–	63±4	65±3
**Aortic valve area index, cm^2^/m^2^**	–	–	0.36±0.02	0.33±0.01
**LVEDDI, mm/m**	30.9±1.0	29.8±0.8	32.2±0.7	30.5±0.8
**IVSTI, mm/m**	7.0±0.3	6.5±0.4	9.3±0.4 ***	9.0±0.5 **
**PWTI, mm/m**	6.0±0.3	5.7±0.3	7.8±0.2 ***	8.1±0.4 ***
**LV ejection fraction, %**	61±2	63±3	61±3	61±3
**LVEDr/PWT**	2.7±0.2	2.7±0.1	2.1±0.1 **	2.0±0.1 ***
**LVMI, g/m^2.7^**	53.7±3.6	46.5±4.0	84.3±4.3 ***	76.2±5.5 ***

LVEDDI: Left ventricular end-diastolic diameter index to height; IVSTI: Interventricular septum thickness index to height; PWTI: Posterior wall thickness index to height; LVEDr/PWT: Left ventricular end-diastolic radius to posterior wall thickness ratio; LVMI: Left ventricular mass index to height^2.7^

* p<0.05;

** p<0.01;

*** p<0.001 (two tailed unpaired *t* test versus same gender control patients).

### Echocardiographic Data

**Figure 1:**
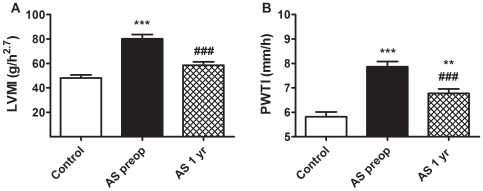
Evolution of left ventricucular mass and posterior wall thickness after aortic valve replacement. Left ventricular mass (in grams) indexed to the height (in meters) of the patient^2.7^ (LVMI) (**A**) and posterior wall thickness (in mm) indexed to the height (in meters) (**B**) in control volunteers, and in AS patients preoperatively and one year after valvular replacement. Results are expressed as mean±SEM. *** *p*<0.001 and ** *p*<0.01 *vs* healthy controls; ### *p*<0.001 *vs* preoperative values (one-way ANOVA and Bonferroni's post-hoc test).

Men and women with AS exhibited no significant difference in aortic valve gradients, or in mean aortic valve area index to body surface area (Table 1), although men displayed a larger crude aortic valve area (0.66±0.03 *vs* 0.55±0.02 cm^2,^ *p*<0.01). AS patients of both genders, compared to controls, showed significantly greater indexed IVST and PWT, and lower LVEDr/PWT ratio. No significant difference in LVEF was found between groups. The AS cohorts showed a significant increase in LVMI, compared with controls (Fig. 1A). One year after surgery, LVMI is normalized but PWTI reduction, albeit significant, did not reach the values of healthy controls (Fig. 1B).

### AS Patients Show Increased Plasma Levels of TGF-β1 in Comparison with Healthy Controls

**Figure 2:**
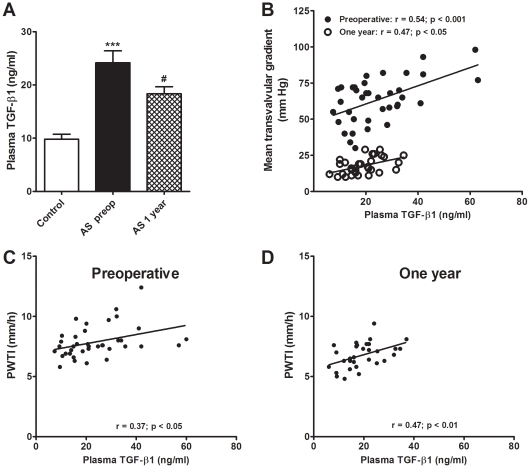
Pre- and one year postoperative plasma levels of TGF-β1 and their relationships with echocardiographic parameters. **A**: Plasma TGF-β1 levels in control volunteers, and in AS patients preoperatively and one year after valvular replacement. Results are expressed as mean±SEM. *p<0.05 and ***p<0.001 *vs* control volunteers (one-way ANOVA followed by Bonferroni's test). **B**: Regression lines showing, in AS patients, the correlation between plasma TGF-β1 concentrations and the mean transvalvular gradients preoperatively (•) and, one year after aortic valve replacement (○), with the residual mean transprosthetic gradients. (Pearson's regression analysis). **C** and **D**: Regression lines showing, in AS patients, the correlation between plasma TGF-β1 concentrations and posterior wall indexed to the height (PWTI) preoperatively (C) and one year after aortic valve replacement (D). (Pearson's regression analysis).

As shown in Figure 2A, AS patients show significantly higher preoperative plasma levels of TGF-β1 when compared with healthy individuals (controls: 9.8±0.9 ng/ml; AS patients: 24.2±2.2 ng/ml). Plasma levels of TGF-β1 determined in peripheral venous blood did not differ significantly from levels measured in the coronary sinus (19.7±3.4 ng/ml). One year after surgery, TGF-β1 plasma levels experiment a significant reduction when compared with preoperative values (AS patients, 1 year after surgery: 18.4±1.3 ng/ml), but do not reach the values of healthy controls (Fig. 2A).

### Plasma Levels of TGF-β1 Are Positively Correlated to Mean Gradients and with Hypertrophy-Related Echocardiographic Parameters in AS Patients

A significant and positive correlation between peripheral TGF-β1 plasma levels and mean aortic transvalvular gradients (Fig. 2B) was evident in AS patients. Four months (data not shown) and one year after surgery, plasma TGF-β1 levels still maintain the positive correlation with the residual mean transprosthetic gradients (Fig. 2B).

Plasma TGF-β1 levels, both preoperatively and 1 year after surgery (Figs. 2C and D), display a significant and direct correlation with PWTI in all AS patients. In addition, a relationship between preoperative TGF-β1and LVMI (r = 0.72, p<0.01) and IVSI (r = 0.67, p<0.01) was evident only in AS women.

### TGF-β1 Plasma Levels Are Positively Correlated with LV Myocardial Expression Levels of Genes Encoding Effectors of the TGF-β1 Signalling Pathways, and with TGF-β1 Target Genes, Encoding Remodelling-Related Proteins

**Figure 3:**
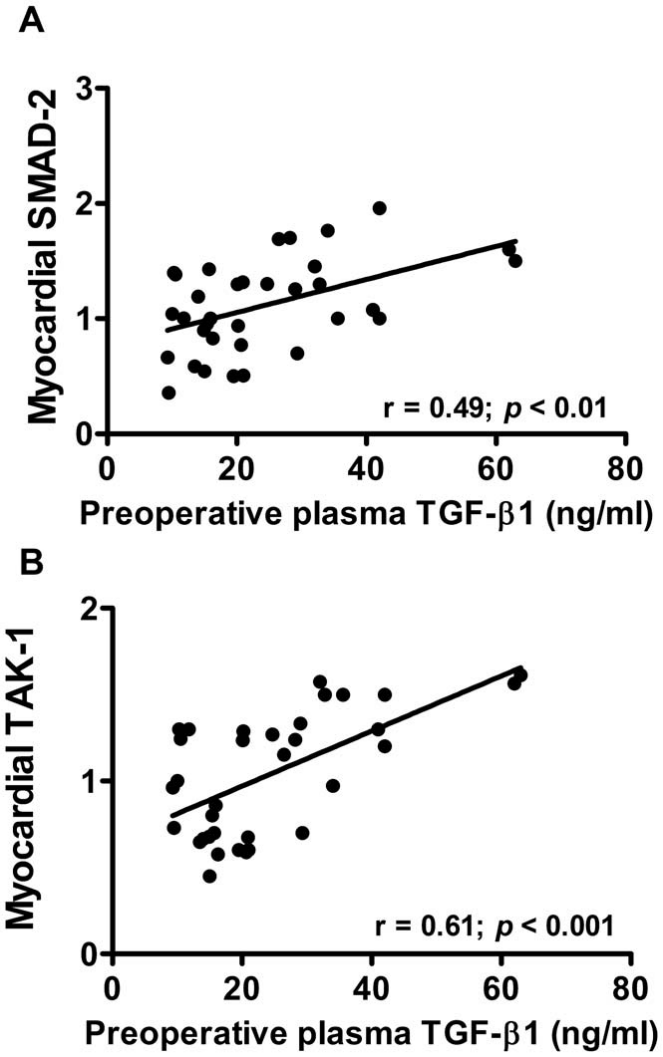
Relationships between circulating TGF-β1 and myocardial gene expression of its canonical and non-canonical effectors. Regression lines show the positive correlation between TGF-β1 plasma levels and LV myocardial mRNA expression of SMAD-2 (**A**) and TAK-1 (**B**) in AS patients. The relative mRNA expression was normalized *vs* the housekeeping gene, ribosomal subunit 18S, and multiplied by 10^5^. (Pearson's regression analysis).

**Figure 4:**
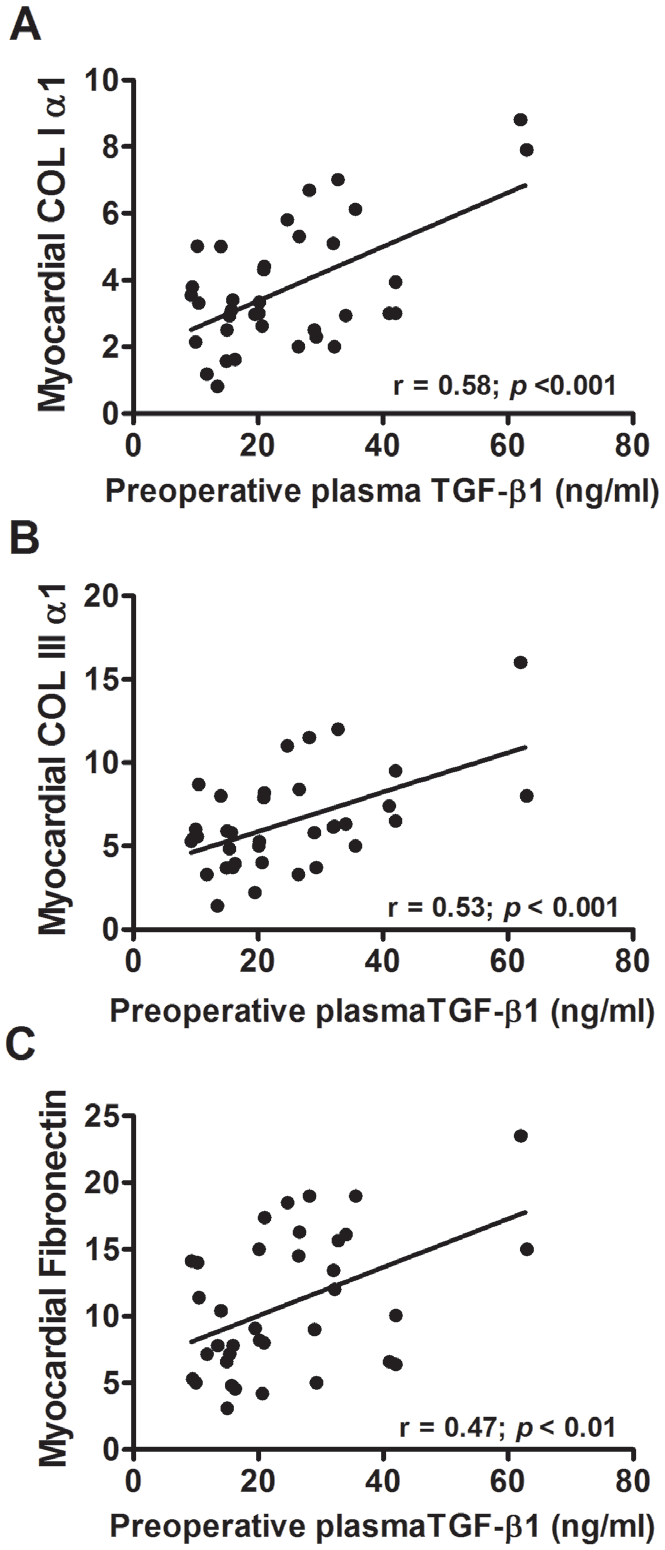
Relationships between circulating TGF-β1 and myocardial gene expression of its target genes of ECM remodeling. Regression lines show the positive correlation between preoperative plasma levels of TGF-β1 and myocardial mRNA expression levels of **A**: collagen I (COLIα1), **B**: collagen III (COLIIIα1) and **C**: fibronectin in AS patients. The relative mRNA expression was normalized *vs* the housekeeping gene, ribosomal subunit 18S, and multiplied by 10^5^. (Pearson's regression analysis).

**Figure 5:**
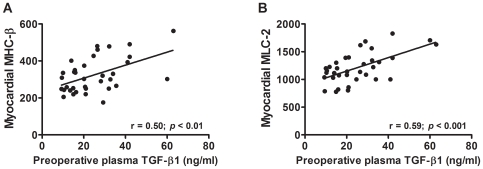
Relationships between circulating TGF-β1 and myocardial gene expression of sarcomeric target genes. Regression lines show the positive significant correlation between preoperative plasma levels of TGF-β1 and myocardial mRNA expression levels of β-myosin heavy chain (**A**) and myosin light chain-2 (**B**), in AS patients. The relative mRNA expression was normalized *vs* the housekeeping gene, ribosomal subunit 18S, and multiplied by 10^5^. (Pearson's regression analysis).

Preoperative plasma levels of TGF-β1 in AS patients sustain a significant positive correlation with LV myocardial SMAD-2 and TAK-1 transcript levels (Fig. 3). The myocardial expression of genes regulated by TGF-β1, such as the ECM elements collagen I, collagen III and fibronectin (Fig. 4) and the sarcomeric proteins myosin light chain-2 and β-myosin heavy chain (Fig. 5), are also directly correlated with plasma TGF-β1 concentrations.

To ascertain whether the myocardium was the main source of plasma TGF-β1 in AS patients, the relationship between plasma and myocardial transcript levels of the cytokine was tested. No significant correlation between both parameters was evidenced (r = 0.27; p = 0.1).

### Mice Subjected to Pressure Overload Feature the Same Response to Plasmatic TGF-β1 than AS Patients

**Figure 6:**
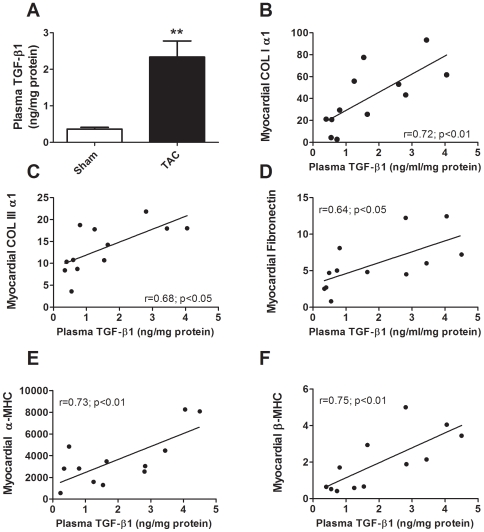
Circulating TGF-β1 in pressure overloaded mice and its relationships with mRNA expression of ECM and sarcomeric remodeling proteins. **A**: Plasma TGF-β1 levels in sham (n = 6) and pressure overloaded mice (n = 12). Results are means±SEM. **p<0.01 *vs* sham controls (Student's t test). **B to F**: Regression lines showing, in 1, and 4 wk TAC mice, the correlation between plasma TGF-β1 concentrations and myocardial mRNA expression levels of **B**: Collagen I (COLIα1), **C**: Collagen III (COLIIIα1), **D**: Fibronectin, **E**: α-myosin heavy chain and **F**: β-myosin heavy chain. The relative mRNA expression was normalized *vs* the housekeeping gene, ribosomal subunit 18S, and multiplied by 10^5^. (Pearson's regression analysis).

As shown in figure 6, plasma levels of TGF-β1 are significantly higher in TAC mice than in sham controls (sham: 0.36±0.05 *vs* TAC: 2.44±0.43; p<0.05), and maintain a significant and positive correlation with myocardial mRNA levels of collagens I and III, fibronectin, β-myosin heavy chain, and with α-myosin heavy chain, which is the predominant isoform in rodents. A positive correlation between plasma TGF-β1 and heart weight index at sacrifice (normalized to body mass) was also evidenced (r = 0.56; p<0.05).

## Discussion

In the present study, we show that preoperative peripheral venous TGF-β1 plasma levels appear increased in AS patients compared with age-matched healthy individuals. Few days after surgery, some AS patients show a transient increase in TGF-β1 levels (data not shown), probably in relation with surgical wound healing processes. During postdischarge follow-up, plasma TGF-β1 recedes significantly, although on the average values do not normalize during the subsequent 12 months. This finding agrees with the well documented notion that reverse LV remodeling after aortic valve replacement in AS patients starts immediately after operation [12], but progresses at a slow pace taking several years to be completed, if ever [13].

A remarkable observation is that, before surgery, plasma levels of TGF-β1 were proportional to the degree of pressure overload the LV of AS patients had to bear, suggesting a link between the severity of the pathology and the release of TGF-β1 to the plasma. Furthermore, 4 months (data not shown) and 1 year after aortic valve replacement, TGF-β1 plasma levels still maintain a significant positive correlation with the residual transprosthetic gradients, so that these two variables still follow a parallel evolution after removing the hemodynamic burden. As to echocardiographic hypertrophy parameters is concerned, we observed that preoperative PWTI kept a close relationship with plasma TGF-β1 in all AS patients, and with LVMI and IVSTI in AS women. One year after valve replacement, this relationship persists with PWTI for the complete cohort. These results would indicate that there is an association of TGF-β1 blood concentration with functional and anatomical parameters reflecting the myocardial changes under pressure overload, so that its levels vary in parallel with changes in the above parameters after valvular replacement.

The key role for myocardial TGF-β1 in the regulation of cardiomyocyte hypertrophic growth and ECM deposition during remodeling following pressure overload is well documented in the experimental animal [3], [4] and in a few reports in human related pathologies [8], [9]. The available experimental data reinforce the view that TGF-β1, released by cardiomyocytes [10] and fibroblasts [11], exerts its effects by acting in an autocrine and/or paracrine manner. In the human scenario, circumstantial evidence suggestive for a contribution of TGF-β1 from a plasmatic source to pressure overload myocardial remodeling was provided by some reports showing that TGF-β1 plasma levels are increased in hypertensive patients, with or without metabolic syndrome [14] with target organ damage, including heart hypertrophy [15], [16]. However, the absence in these studies of data from myocardial biopsies precludes the establishment of a cause-effect relationship between plasma TGF-β1 and LV remodeling. Matt and colleagues [17] reported recently that, in an experimental mutant mouse model of Marfan syndrome that recapitulates the cardiovascular involvement of the disease, circulating levels of TGF-β1 were significantly increased and correlated with the aortic root diameter. They also suggested the potential value of plasma level of this cytokine in Marfan patients, as a marker of disease progression and/or therapeutic response to treatments [17]. Here, the myocardial transcriptional changes, hallmarks of AS, seem to be under the influence of circulating TGF-β1, as suggested by the direct correlation between preoperative plasma levels of TGF-β1 and LV expression of genes encoding ECM elements (collagens I and III and fibronectin) as well as sarcomeric elements (β-myosin heavy chain and myosin light chain-2). Further, these relationships were absent in the myocardium from a cohort of 16 patients operated of cardiac pathologies, with no LV pressure or volume overload (data not shown).

Inferences from the clinical study are limited by the inherent heterogeneity of patients that exhibit many conditions known to have an influence on LV remodeling (systemic hypertension, obesity, diabetes, medical treatments, lack of genetic homology, unknown duration of the pressure overload situation, etc) and could contribute to weaker, albeit significant, linearity of the correlations. To incorporate further evidence, and using a reverse translational approach, we also showed that this blood borne mediated mechanism is operative in mice subjected to TAC, as plasma TGF-β1 and myocardial gene expression of remodeling elements are linked by a relationship similar to the one found in human patients. Important inferences from these results are that, in LV pressure overload, circulating TGF-β1 contributes to the progression of myocardial hypertrophy and fibrosis and, in patients with valvular aortic stenosis, mirrors the myocardial transcriptional activity.

The direct correlation between plasma levels of TGF-β1 and myocardial expression of genes encoding SMAD-2 and TAK-1 reinforces the hypothesis that LV remodeling is dependent not only on local, but also on circulating TGF-β1 mediated mechanisms, involving both canonical and non-canonical downstream effectors, and suggests that the intracellular effectors of TGF-β1 signaling are under the transcriptional influence of circulating TGF-β1.

Our experimental approach does not allow ascertaining the full spectrum of plasmatic TGF-β1 sources, which can be numerous [18]. Indeed, the first candidate to consider is the stressed myocardium. However, in our cohort of patients, the absence of a significant relationship between TGF-β1 mRNA expression in LV myocardial and its concentration in neither peripheral nor coronary sinus blood (data not shown) precludes a major contribution of myocardial tissue to circulating TGF-β1 in this pathology. In addition, coronary sinus and peripheral venous blood concentrations of TGF-β1 were similar, even though the coronary sinus blood has been reported to reflect better the myocardial status of some remodeling-related biomarkers [19], [20]. Should the myocardium be the main source of plasma TGF-β1, then a positive gradient from its concentration in coronary sinus blood towards peripheral vein blood would have been evident. Furthermore, in TAC mice, myocardial and plasmatic TGF-β1 did not correlate either (data not shown). Thus, our data suggest that other sources apart from the myocardium may have additionally contributed to an excess plasma TGF-β1 in response to pressure overload. Putative contributors could be, among others, the stressed endocardium [21], the circulating blood cells activated by shear stress in the restricted aortic orifice region [22] or, in AS patients, the sclerosed aortic valve tissue [23].

In summary, the present study provides new evidence on the involvement of a circulating TGF-β1-mediated mechanism in the excessive deposition of ECM elements and hypertrophic growth of cardiomyocytes in response to pressure overload. Given the moderate power of the association between circulating TGF-β and LV remodeling variables, a single elevated value of this cytokine in patients with aortic stenosis would be of limited help in surgical decision taking. However and according to our results, an escalating time course of the circulating cytokine may reflect progressive myocardial hypertrophy and fibrosis and could be an additional argument for surgery in some asymptomatic or poorly symptomatic patients. Further clinical longitudinal studies are warranted in larger patient populations to confirm the relative merit of circulating TGF-β as a clinically useful biomarker of LV remodeling.

## Materials and Methods

### Ethics Statement

The study followed the Declaration of Helsinki guidelines for investigation on human subjects. The University Hospital Valdecilla Institutional Ethics and Clinical Research Committee approved the study, and all patients gave written informed consent.

The use of mice in the study was approved by the University of Cantabria Institutional Laboratory Animal Care and Use Committee and carried out in accordance with the Declaration of Helsinki and the European Communities Council Directive (86/609/EEC).

### Patients

The study was performed on myocardial and plasma samples obtained prospectively from 39 patients with severe AS undergoing cardiac surgery (21 men and 18 women). Patients with aortic or mitral regurgitation greater than mild and patients with major coronary stenosis greater than 50% were ineligible for the study. The control group was constituted by 27 healthy volunteers that were recruited for their ages similar to those of AS patients and lack of previous cardiac disease. The clinical and echocardiographic characteristics of both AS and control groups are shown in Table 1.

### Echocardiography

A two-dimensionally guided M-mode transthoracic echocardiogram was performed (Philips-Hewlett Packard, IE 33) in AS patients preoperatively, prior to hospital discharge after valve replacement, and four months and one year postoperatively. In the control individuals, a similar study was performed at the time of blood sampling.

M-mode tracings were digitally recorded and analyzed. Internal LVEDD and LVESD diameters, IVST, and PWT were recorded. LV dimensions and wall thicknesses were indexed to the height of the patient. Relative LV end diastolic radius was expressed as the ratio between LV cavity end-diastolic radius and PWT. LVEF was calculated using Quiñones formula, and LVM according to Devereux formula [24], indexed to height of the patient to the 2.7^th^ power [25] and expressed in g/m^2.7^.

### Blood Sampling and Determination of TGF-β1 Plasma Levels

Samples were collected from peripheral venous blood of healthy volunteers and AS patients. In the latter group, samples were drawn 24 h preoperatively, at operation from the coronary sinus and, in the postoperative period, at the time of the echocardiographic studies. To minimize platelet degranulation [18], blood was drawn from an antecubital vein without tourniquet, using a syringe with a wide-gauge needle and then gently transferred to a collection tube containing EDTA. Within 15 minutes of blood collection, plasma was separated by centrifugation at 1000×g for 30 minutes. Plasma aliquots were stored at −80°C until analysis. Total TGF-β1 plasma levels were determined by ELISA (Biotrak Easy ELISA, GE Healthcare), following the manufacturer's instructions.

### Myocardial Sampling and Quantitative PCR

During surgery, myocardial tru-cut needle biopsies (4–10 mg) were taken from the lateral LV wall and immediately frozen in liquid nitrogen.

Total myocardial RNA was retro-transcribed into cDNA. Quantitative PCR was conducted using specific TaqMan assays (Applied Biosystems). The expression of genes encoding TGF-β1, collagen I, collagen III, fibronectin, myosin light chain-2 and β-myosin heavy chain was normalized to the housekeeping gene, ribosomal subunit 18S, measured in parallel in each sample. Duplicate transcript levels were determined in three independent experiments. Results are expressed as 2^(housekeeping Ct-problem Ct)^ multiplied by 10^5^, being Ct the cycle threshold.

### Pressure Overload Studies in Mice

The experiments were performed in adult (16–20 weeks old) C57BL/6 mice, housed in a room kept at 22°C with 12∶12 h light/dark cycle, and provided with food and water *ad libitum*.

Pressure overload was induced by controlled constriction of the transverse aortic arch (TAC) as described previously [26]. Briefly, mice were anesthetized by intraperitoneal injection of ketamine (10 mg/kg) and xylazine (15 mg/kg). The aorta was constricted at the mid arch level with a 7/0 polypropylene ligature using as calibrator a blunted 27-gauge (0.41 mm OD) needle, reaching approximately a 70% stenosis in diameter. Mice were sham operated (n = 6) or subjected to TAC for 1 or 4 weeks (n = 6 per group). After completing their follow-up, mice were anesthetized with ketamine and xylazine, blood samples were collected from the retroorbitary sinus, and LV samples were immediately frozen in liquid nitrogen for RNA extraction. Plasma levels of TGF-β1 were determined by ELISA, and myocardial expression of genes encoding TGF-β1, collagen I, collagen III, fibronectin, α-myosin heavy chain and β-myosin heavy chain by real time PCR, as described below for patients.

### Statistics

GraphPad Prism 5.01 software package was used. Values are reported as means±S.E.M. Student's *t-*test was used to assess differences between means of continuous variables. Multiple comparisons were performed using the one-way ANOVA followed by Bonferroni's test. Categorical data were compared by Fisher's exact test. Pearson's regression analysis was used to detect correlations between plasma and gene expression levels and/or echocardiographic parameters. A *p* value<0.05 was considered significant.
